# Periodontitis and Oral Pathogens in Colorectal Cancer: A Systematic Review, Meta-Analysis, and Trial Sequential Analysis

**DOI:** 10.3390/dj13120595

**Published:** 2025-12-12

**Authors:** Luis Chauca-Bajaña, Andrea Ordoñez Balladares, Alejandro Ismael Lorenzo-Pouso, Rosangela Caicedo-Quiroz, Rafael Xavier Erazo Vaca, Rolando Fabricio Dau Villafuerte, Yajaira Vanessa Avila-Granizo, Carlos Hans Salazar Minda, Miguel Amador Salavarria Vélez, Byron Velásquez Ron

**Affiliations:** 1 College Dentistry, University of Guayaquil, Guayaquil 090101, Ecuador; 2 Universidad Bolivariana del Ecuador, Durán 092406, Ecuador; 3 Oral Medicine, Oral Surgery and Implantology Unit (MedOralRes), Faculty of Medicine and Dentistry, University of Santiago de Compostela, 15782 Santiago de Compostela, Spain; 4 Carrera de Odontología, Universidad de Las Américas (UDLA), Department Prosthesis Research, Quito 170102, Ecuador

**Keywords:** colorectal neoplasms, *Fusobacterium nucleatum*, gastrointestinal microbiome, oral microbiome, *Porphyromonas gingivalis*, periodontitis

## Abstract

**Background**: Periodontitis and oral dysbiosis have been linked to systemic inflammation and carcinogenesis. Among oral pathogens, *Porphyromonas gingivalis* (Pg) and *Fusobacterium nucleatum* (Fn) are biologically plausible contributors to colorectal cancer (CRC) via inflammatory and immunomodulatory pathways. However, the magnitude and consistency of these associations remain uncertain. **Objective**: To evaluate whether periodontitis and key oral pathogens are associated with CRC risk and prognosis through a systematic review, meta-analysis, and trial sequential analysis (TSA). **Methods**: We searched PubMed, Scopus, and Web of Science from inception to December 2024 following PRISMA 2020. Eligible observational studies assessed periodontitis exposure or detection of oral bacteria in relation to CRC incidence or survival. Effect estimates (RRs/HRs) were log-transformed and pooled using random-effects models; heterogeneity was quantified with I^2^. TSA was conducted to appraise information size and the stability of the primary association. Risk of bias was evaluated with ROBINS-I/QUIPS as appropriate. PROSPERO: CRD420251168522. **Results**: Five studies evaluating periodontitis/oral-pathogen exposure and CRC incidence yielded a 70% higher risk (HR = 1.70; 95% CI: 1.33–2.19; I^2^ = 0%). Detection of Fn was associated with approximately threefold higher risk of CRC (RR = 3.20; 95% CI: 1.76–5.82; *p* < 0.001). Pg presence was linked to worse overall survival (HR ≈ 2.4; *p* < 0.01). TSA suggested that the accrued evidence for the primary incidence association is likely sufficient to reduce random errors; nevertheless, interpretability is constrained by the small number of observational studies and between-study differences in exposure and outcome ascertainment. **Conclusions**: Current evidence indicates that periodontitis and oral pathogens—particularly Fn and Pg—are significantly associated with CRC development and progression. These findings support the clinical relevance of the oral–gut axis and underscore oral health as a potentially modifiable factor in cancer prevention. Further large, well-designed prospective cohorts and mechanistic studies are warranted to strengthen causal inference.

## 1. Introduction

Colorectal cancer (CRC) remains one of the greatest challenges in modern oncology [1]. It accounts for approximately 10% of all new cancer diagnoses and 9% of cancer-related deaths worldwide [2,3,4]. Despite advances in prevention and treatment, its incidence continues to rise, particularly in developing countries [5].

CRC originates from the deregulated proliferation of epithelial cells in the colon or rectum, driven by a combination of genetic, inflammatory, environmental, and microbial factors [6,7]. Over the last decade, the notion that the intestinal microbiome is not a mere bystander but an active player in colorectal carcinogenesis has gained prominence [8,9]. Intestinal dysbiosis can promote chronic inflammation, damage the epithelial barrier, modulate mucosal immunity, and generate metabolites with genotoxic effects [10,11,12]. Together, these mechanisms foster a tumor-prone microenvironment conducive to cancer initiation and progression. More recently, the oral microbiota has been recognized as potentially playing a similar role [13]. Each day, millions of oral bacteria are swallowed and reach the gastrointestinal tract; under chronic periodontitis, this microbial flux may include highly virulent pathogens capable of colonizing the colon and altering its microenvironment [14,15]. This connection between the mouth and the gut, known as the oral–gut axis, has opened new perspectives on the relationship between periodontal health and colorectal carcinogenesis [16]. Among the most investigated microorganisms is Fn, whose presence has been detected in tumor tissue and feces of patients with CRC. This pathogen not only increases tumor multiplicity but also promotes metastasis, local inflammation, and chemoresistance [17,18,19,20]. In parallel, the periodontal pathogen Pg has emerged as a new candidate of oncologic interest. Recent clinical studies have identified Pg in fecal and tumor samples from patients with CRC, associating it with worse survival and tumor progression [21,22]. Experimentally, it has been shown to activate the NOD-like receptor family pyrin domain–containing 3 (NLRP3) inflammasome, stimulate proliferative pathways such as the mitogen-activated protein kinase/extracellular signal–regulated kinase (MAPK/ERK) pathway, and modulate immune responses via invariant natural killer T (iNKT) cells, thereby promoting an inflammatory and immunosuppressive tumor microenvironment [23,24,25]. Meanwhile, periodontitis—a chronic inflammatory disease of the periodontium—has been consistently linked to an increased risk of colorectal cancer [26,27]. However, differences in diagnostic methods, molecular techniques, and confounder adjustment have yielded heterogeneous results across studies, complicating the joint interpretation of findings. In this context, our aim is to systematize and quantify the relationship between periodontitis, oral pathogens (especially Pg and Fn), and CRC through a systematic review, meta-analysis, and trial sequential analysis. We seek to answer two clinical questions: (i) whether oral exposure (periodontitis or pathogen detection) is associated with increased CRC risk/incidence; and (ii) whether tumor or fecal detection of these bacteria is related to worse survival. Our ultimate goal is to provide reproducible, clinically useful evidence that bridges laboratory and clinic, and helps profile risk and prognostic biomarkers to inform personalized prevention and management strategies.

## 2. Materials and Methods

### 2.1. Protocol and Registration

**Figure 1:**
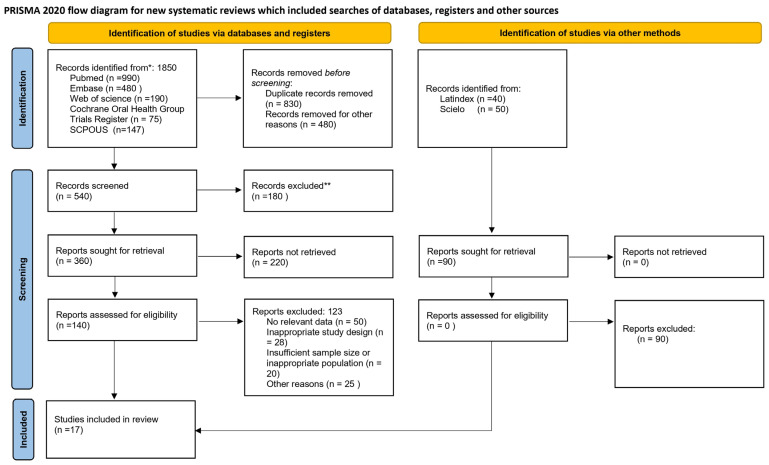
Flowchart of selected studies.* “Records identified” refers to the total number of references retrieved from each database before removing duplicates or other previously excluded records. ** “Records excluded” refers to studies discarded during title and abstract screening because they did not meet the PICO criteria, were not observational human studies, did not evaluate periodontitis or oral pathogens, or did not report data rela-ted to colorectal cancer outcomes.

A protocol specific to this systematic review and meta-analysis was designed in strict accordance with the Preferred Reporting Items for Systematic Reviews and Meta-Analyses (PRISMA 2020) guidelines (Figure 1, Table S1) [28]. The protocol was prospectively registered in the International Prospective Register of Systematic Reviews (PROSPERO) under the identifier CRD420251168522. Registration aims to ensure transparency of the process, minimize risk of bias, and provide methodological traceability.

### 2.2. PICO Question

In adults with periodontitis, is there a higher risk of developing colorectal cancer and/or greater detection of periodontopathogenic oral bacteria (*Fusobacterium nucleatum*, *Porphyromonas gingivalis*, *Tannerella forsythia*, among others) in colorectal tumor tissue, the fecal microbiome, or biological fluids, compared with adults without periodontitis or without these pathogens? Likewise, is the presence of these bacteria associated with worse prognosis in terms of survival?

Population (P): adults enrolled in observational studies (cohorts, case–control, population-based studies) and human molecular studies. Intervention/Exposure (I): clinical or radiographic diagnosis of periodontitis and/or detection of associated oral bacteria (*F. nucleatum*, *P. gingivalis*, *T. forsythia*, among others) in tissue, biological fluids, or the fecal microbiome using molecular techniques (PCR, 16S rRNA sequencing, metagenomics). Comparison (C): adults without periodontitis or without a pathogenic oral microbiome. Outcome (O): The primary outcome was the risk of incident colorectal cancer (CRC) associated with periodontitis or periodontal pathogens. Secondary outcomes were: (i) prognosis among CRC patients (overall survival [OS] and recurrence-free/progression-free survival [RFS/PFS]) according to periodontal status or pathogen load; and (ii) microbiological outcomes (prevalence and/or relative abundance of periodontal pathogens in CRC patients versus non-CRC controls). We did not combine “tumor detection” or fecal/biomarker detection outcomes with incidence risk, as these represent distinct constructs. Diagnostic-accuracy metrics (e.g., sensitivity/specificity, AUC) were analyzed separately or narratively.

### 2.3. Database Search Strategy and Screening

Rayyan QCRI (Qatar Computing Research Institute, Doha, Qatar) was used to organize, screen, and select potentially eligible articles. The search strategy comprised a comprehensive review of multiple databases: MEDLINE (PubMed), EMBASE (Ovid), Web of Science, Scopus, the Cochrane Library, ClinicalTrials.gov, and the five WHO regional databases (AIM, LILACS, IMEMR, IMSEAR, WPRIM), as well as the Conference Proceedings Citation Index. For each database, a tailored combination of keywords and controlled vocabulary (e.g., MeSH/Emtree) was developed and refined to maximize sensitivity and specificity. The core search model included the following terms: periodontitis, periodontal disease, gingival disease, colorectal cancer, colon neoplasms, rectal neoplasms, oral microbiome, *Fusobacterium nucleatum*, *Porphyromonas gingivalis*, *Tannerella forsythia*, and Prevotella intermedia. Boolean operators (AND/OR) were applied as appropriate to combine terms.

### 2.4. Eligibility Criteria

**Inclusion**

- Observational studies (cohorts, case–control, population-based) reporting an association between periodontitis and colorectal cancer.
- Human molecular studies identifying periodontopathogenic bacteria (*F. nucleatum*, *P. gingivalis*, *T. forsythia*, among others) in tumor tissue, the fecal microbiome, or biological fluids of patients with colorectal cancer.
- Adults (≥18 years).
- Languages: English.
- Published from 2000 onward.

**Exclusion**

- Animal or in vitro studies.
- Systematic reviews, narrative reviews, editorials, letters, comments.
- Studies not reporting quantifiable risk data (OR, RR, HR, incidence) or confirmed bacterial presence.
- Studies that do not clearly distinguish periodontitis from healthy controls, or an oral microbiome from the general intestinal microbiome.

### 2.5. Study Selection Process and Data Extraction

Two independent reviewers (L.C.H. and B.V.R.) systematically conducted the search, selection, and screening of identified studies. In the first stage, titles and abstracts of all retrieved records were assessed. Potentially eligible articles were then reviewed in full text to confirm final inclusion. Discrepancies were resolved by consensus and, when necessary, with the involvement of a third reviewer (A.O.B.), who was blinded to the main hypothesis to maintain objectivity and reduce selection bias. Data extraction was performed using a standardized form specifically designed for this review. The following variables were collected from each study: first author, year of publication, country or cohort, study design, total sample size, number of cases and controls, diagnostic criteria for periodontitis or oral exposure, oral bacteria analyzed (*Fusobacterium nucleatum* and *Porphyromonas gingivalis*), microbiological detection method (qPCR, 16S rRNA sequencing, NGS, immunohistochemistry, or ELISA), sample type (tumor tissue, saliva, stool, or serum), clinical and histopathological characteristics of colorectal cancer, oncologic diagnostic method (histology, national registries, or ICD codes), reported effect measure (HR, OR, or RR), adjustment for confounders, follow-up time (when applicable), and main findings.

The characteristics and findings of the included studies are summarized in Table 1, Table 2 and Table 3, which group the evidence thematically as follows:

- Table 1 presents studies evaluating the overall relationship between periodontitis or exposure to oral pathogens and colorectal cancer risk, including prospective cohorts and case–control studies.
- Table 2 includes articles focused on *Fusobacterium nucleatum*, highlighting its tumor frequency, molecular associations (such as MSI or BRAF mutations), and potential prognostic value.
- Table 3 summarizes studies analyzing the involvement of *Porphyromonas gingivalis* in colorectal carcinogenesis and its impact on survival, in both clinical and experimental settings.

### 2.6. Risk of Bias Assessment

Risk of bias assessment. Two independent reviewers (L.C.H., B.V.R.) evaluated risk of bias by study type, with disagreements resolved by consensus and, when necessary, a third reviewer (A.O.B.). For observational studies estimating CRC risk from periodontitis/oral exposure (Table 4), we used ROBINS-I, assessing confounding, selection, exposure classification, missing data, outcome measurement, and selective reporting (judgments: low/some concerns/high). For detection studies of *Fusobacterium nucleatum* and *Porphyromonas gingivalis* (Table 5 and Table 6), we applied QUADAS-2 (patient selection, index test, reference standard, flow/timing, and applicability). For prognostic analyses (survival vs. bacterial burden in tissue/stool), we used QUIPS (study participation, prognostic factor measurement, confounding, outcome measurement, follow-up, and analysis). Finally, for prevalence estimates of *P. gingivalis*, we used the JBI/Hoy checklist (sampling frame, case definition, measurement method, non-response/missing data, and analysis). Prior to formal assessment, reviewers calibrated their judgments; domain-level decisions and overall ratings were documented in Supplementary Table S1.

### 2.7. Statistical Analysis

For the meta-analyses, we used a quantitative approach based on random-effects models to synthesize evidence from observational studies evaluating the relationship between the presence of *Fusobacterium nucleatum* or *Porphyromonas gingivalis* and clinical outcomes in colorectal cancer. This approach assumes that the true effect sizes may vary across studies, reflecting inherent differences in populations, diagnostic methods, and experimental conditions. Pooled estimates were calculated using the DerSimonian–Laird method and expressed as risk ratios (RR), odds ratios (OR), or hazard ratios (HR) with their corresponding 95% confidence intervals, depending on the metric reported in each study.

Between-study heterogeneity was quantified using the I^2^ statistic and Cochran’s chi-squared (Q) test, with values >50% considered indicative of substantial heterogeneity. When high heterogeneity was detected, potential sources of variability were explored according to study design, sample type (fecal, tumor, or tissue), and bacterial detection method (PCR, qPCR, FISH, or 16S rRNA sequencing). Prediction intervals were also computed to estimate the range within which the results of future similar studies might fall. Publication bias was assessed by visual inspection of funnel plots and, when feasible, by Egger’s test to detect statistical asymmetry. Funnel plots were used qualitatively to identify imbalances in the distribution of studies by effect size and precision, with symmetry suggesting absence of bias. To further determine the robustness and sufficiency of the accumulated evidence, we conducted Trial Sequential Analysis (TSA), applying O’Brien–Fleming boundaries to evaluate whether the available information had reached the required sample size to confirm the findings with a controlled type I error. This integrated framework ensured a rigorous and reproducible statistical treatment of the evidence, supporting the validity and stability of the derived conclusions.

## 3. Results

### 3.1. Risk of Colorectal Cancer in Patients with Periodontitis or Exposure to Oral Pathogens

**Figure 2:**
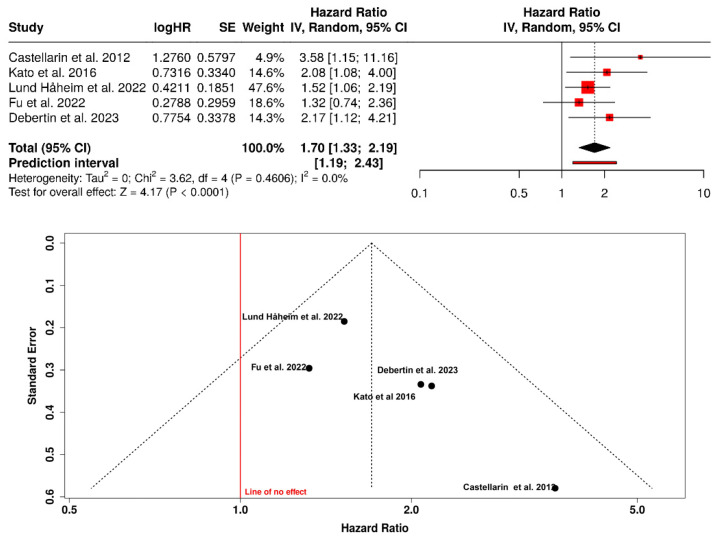
Forest and funnel plots of the association between periodontitis (or exposure to oral pathogens) and colorectal cancer risk [17,29,30,31,32].

The meta-analysis integrated five observational studies (two case–control and three prospective cohort studies) that evaluated the association between periodontitis or exposure to oral pathogens and the risk of colorectal cancer. The main characteristics and quantitative results of these studies are summarized in Table 1. The random-effects analysis showed a 70% increase in the risk of developing colorectal cancer among individuals with a periodontal history or serological evidence of bacterial infection (HR = 1.70; 95% CI: 1.33–2.19; I^2^ = 0%). No significant heterogeneity was observed across studies. The funnel plot displayed a symmetric distribution of points around the pooled effect axis, with no evidence of publication bias. These findings consistently support a potential etiopathogenic role of the oral–gut axis in colorectal carcinogenesis (Figure 2).

**Figure 3:**
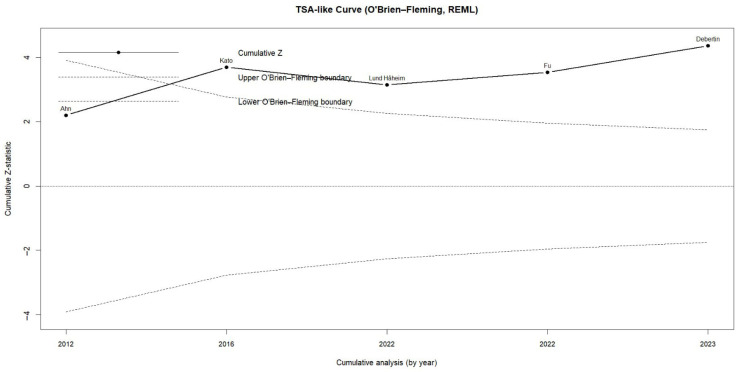
Trial Sequential Analysis (TSA) assessing the cumulative evidence for the association between periodontitis or oral pathogens and colorectal cancer.

Trial sequential analysis (TSA) confirmed the statistical stability of the effect. The cumulative Z-curve crossed the upper O’Brien–Fleming boundary at early stages and remained above the significance threshold throughout the entire analysis period. This pattern indicates that the association between periodontal pathogen infection and colorectal cancer is consistent, stable, and statistically robust, ruling out the possibility that the findings are due to chance (Figure 3).

### 3.2. Porphyromonas Gingivalis and Colorectal Cancer

**Association forest plot (OR, detection in CRC vs. controls)**

**Figure 4:**
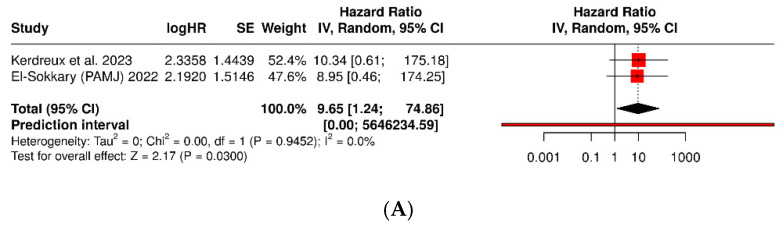

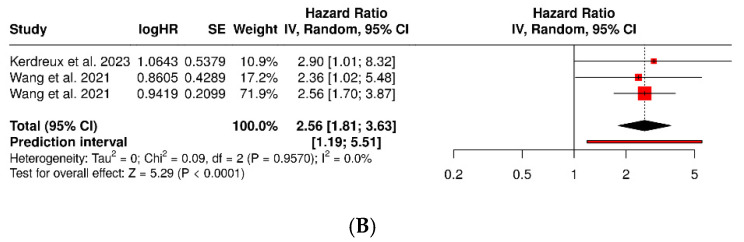
Forest plots of *Porphyromonas gingivalis*: (**A**) association with colorectal cancer diagnosis (OR, detection in stool/tumor vs. controls); (**B**) association with overall survival in CRC (HR) [21,22,42].

Key characteristics of the studies assessing *Porphyromonas gingivalis* and colorectal cancer are summarized in Table 3. The subanalysis of two case–control studies (El-Sokkary 2022 [42]; Kerdreux 2023 [21]) showed that fecal or tumor detection of *Porphyromonas gingivalis* is significantly associated with a diagnosis of colorectal cancer (OR = 9.65; 95% CI: 1.24–74.86; *p* = 0.030; I^2^ = 0%). This implies that patients with colorectal cancer are nearly ten times more likely to have detectable *P. gingivalis* in stool or tissue than controls. Regarding prognosis, three cohorts (Wang 2021 [22], cohorts 1 and 2; Kerdreux 2023 [21]) demonstrated a consistent relationship between tumor or fecal presence of *P. gingivalis* and worse overall survival (HR = 2.56; 95% CI: 1.81–3.63; *p* < 0.0001; I^2^ = 0%) (Figure 4).

**Figure 5:**
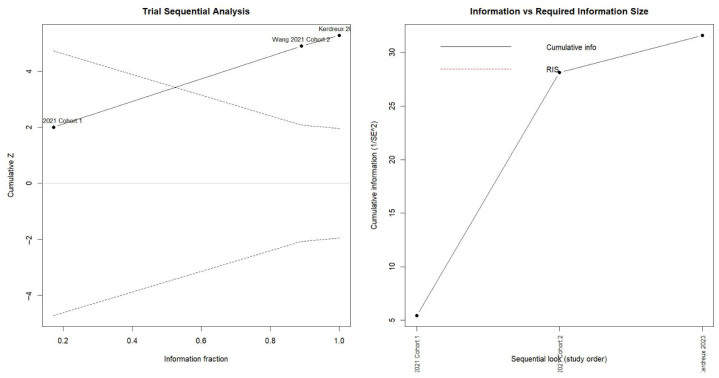
Trial sequential analysis (TSA) of the association between intratumoral *Porphyromonas gingivalis* and overall survival in colorectal cancer [22].

Trial sequential analysis (TSA) showed that the evidence accumulated over time is solid and consistent. The Z-curve crossed the O’Brien–Fleming boundaries and reached the required information size, indicating that the association between intratumoral *Porphyromonas gingivalis* and overall survival in colorectal cancer is conclusive (Figure 5).

**Figure 6:**
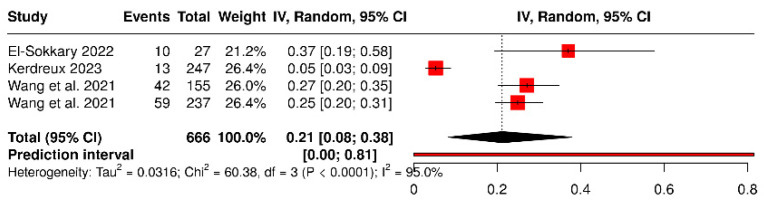
Forest plot of the pooled prevalence of *Porphyromonas gingivalis* in colorectal cancer [21,22,42].

The prevalence meta-analysis (four studies) estimated a pooled proportion of *P. gingivalis* of 21% (95% CI: 8–38%), with high heterogeneity (I^2^ = 95%). This variability is attributable to methodological differences (sample type and detection technique). Even so, the direction of effect was consistent across studies, suggesting that *P. gingivalis* is a frequent component of the colorectal tumor microbiome and a potential marker of poor prognosis (Figure 6).

### 3.3. Fusobacterium Nucleatum and Colorectal Cancer

**Figure 7:**
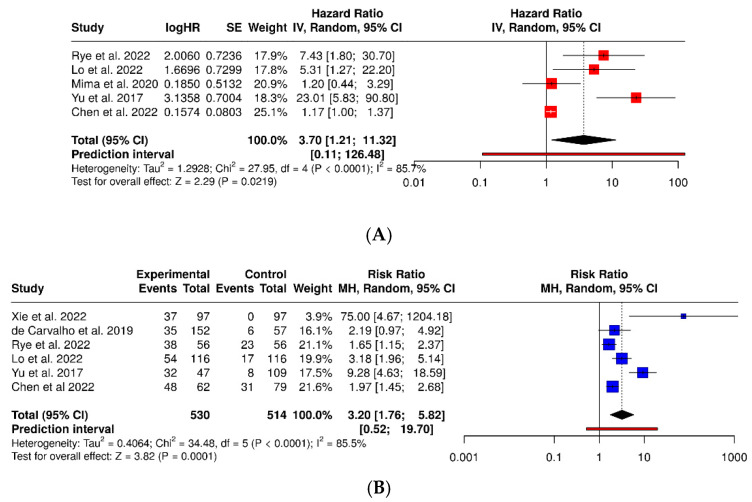
Forest plots of the association between Fusobacterium nucleatum and colorectal cancer risk: (**A**) pooled hazard ratio; (**B**) pooled relative risk [33,35,37,38,39,40,41].

A summary of studies evaluating *Fusobacterium nucleatum* in colorectal cancer is presented in Table 2. The meta-analysis of five observational studies (Yu 2017 [40]; Mima 2020 [39]; Rye 2022 [37]; Lo 2022 [38]; Chen 2022 [41]) showed that the presence of *Fusobacterium nucleatum* is significantly associated with the development of colorectal cancer, with a pooled hazard ratio of 3.70 (95% CI: 1.21–11.32; *p* = 0.0219). Consistently, the forest plot demonstrated a pooled relative risk of 3.20 (95% CI: 1.76–5.82; *p* = 0.0001), indicating that individuals with detectable *F. nucleatum* are more than three times as likely to develop colorectal cancer compared with controls. Despite high heterogeneity (I^2^ = 85.5%), the direction of effect was uniform, supporting the role of *F. nucleatum* as a potential risk biomarker (Figure 7).

**Figure 8:**
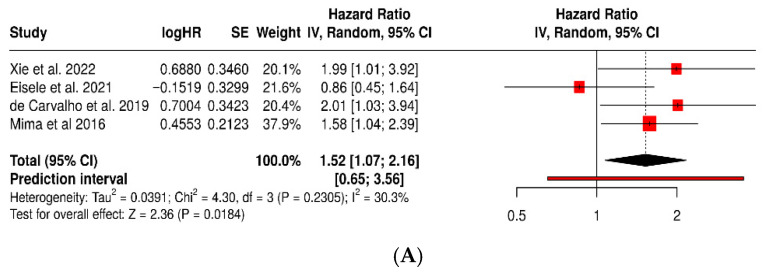

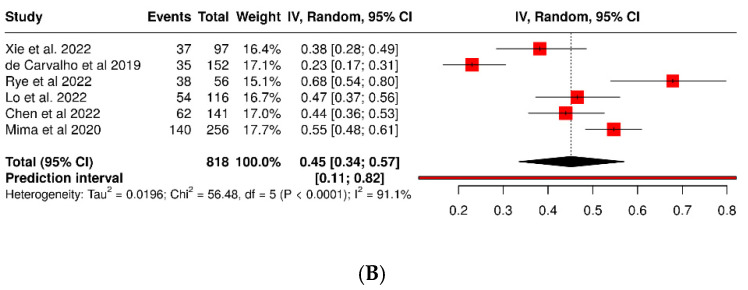
Forest plots of *Fusobacterium nucleatum*: (**A**) association with overall survival in colorectal cancer (HR); (**B**) pooled prevalence in tumor tissue (proportion meta-analysis) [33,34,35,36,37,38,39,41].

In the prognostic analysis, a high intratumoral load of *Fusobacterium nucleatum* was associated with worse overall survival (HR = 1.52; 95% CI: 1.07–2.16; *p* = 0.018; I^2^ = 30.3%), confirming its role as an adverse marker of clinical course. The single-arm meta-analysis estimated a pooled prevalence of 45% (95% CI: 34–57%) in tumor tissues, demonstrating that *F. nucleatum* is a frequent and biologically relevant finding in colorectal cancer (Figure 8).

**Figure 9:**
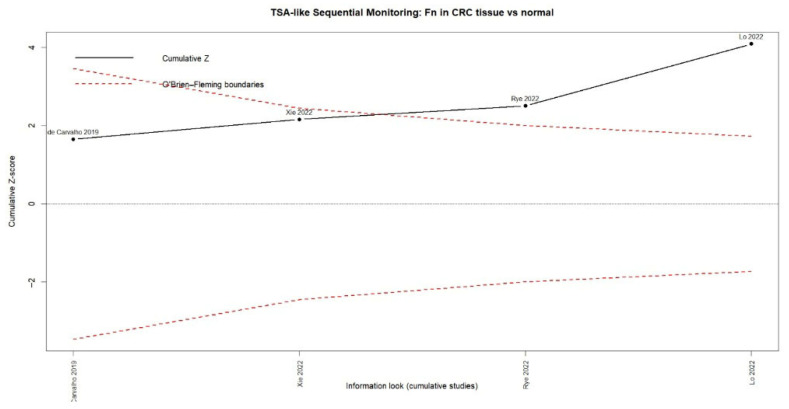
Trial sequential analysis (TSA) of *Fusobacterium nucleatum* detection in colorectal cancer tissue versus normal tissue (cumulative Z-curve with O’Brien–Fleming boundaries) [33,35,37,38].

The corresponding TSA showed that the accumulated evidence has exceeded the O’Brien–Fleming significance limits, indicating that the relationship between *F. nucleatum* and colorectal cancer is supported by statistically robust and sufficient evidence (Figure 9).

## 4. Discussion

The present systematic review and meta-analysis indicate that periodontal disease—characterized by chronic inflammation and oral dysbiosis—and the detection of specific oral pathogens are associated with colorectal cancer (CRC) outcomes. The pooled risk estimate (HR = 1.70; 95% CI: 1.33–2.19) for periodontitis/oral-exposure and incident CRC suggests that the impact of oral health extends beyond the oral cavity and may contribute to a pro-inflammatory systemic milieu. Consistent associations were observed for *Fusobacterium nucleatum* (Fn) with CRC incidence and for *Porphyromonas gingivalis* (Pg) with adverse overall survival, supporting the biological plausibility of an oral–gut axis in carcinogenesis. However, these findings derive largely from observational studies and should not be interpreted as causal. Our results align with prior syntheses reporting strong enrichment of Fn in tumor tissue and worse survival in cases with higher microbial burden. For example, a meta-analysis of 45 studies estimated an odds ratio of ~10 for Fn presence in tumor tissue versus controls and an HR ≈ 1.87 for overall survival with high Fn load [43]. In parallel, recent clinical cohorts report that fecal Pg detection correlates with poorer survival (HR ≈ 2.90) [21]. These patterns suggest that certain oral microorganisms may act as context-dependent modulators of the tumor microenvironment rather than transient bystanders. A plausible explanation for this non-causal presence is that colorectal tumors create conditions that are especially favorable for anaerobic bacteria such as Pg and Fn. Tumor tissue is typically low in oxygen, rich in nutrients, and locally immunosuppressed, which reduces immune clearance and makes these lesions a permissive niche. Both species are also capable of surviving inside host cells, which may further support their persistence once colonization occurs. Pg has been shown to promote the survival of its host epithelial and immune cells by modulating apoptosis and inflammatory signaling, while Fn can attach to and invade tumor cells, allowing intracellular residence. These features help explain why these bacteria are frequently detected in colorectal tumors without implying that they initiate carcinogenesis. Mechanistically, multiple lines of evidence support this interpretation. Fn can adhere to and invade intestinal epithelium via FadA, activating β-catenin signaling and reshaping local immunity [44,45,46,47]. Experimental work further suggests strain-level adaptations that enhance gastric acid tolerance and epithelial adherence, potentially facilitating ectopic colonization [44,48]. In addition to these findings, several basic research studies show that *F. nucleatum* promotes tumor progression through additional mechanisms. These include activation of the TLR4–MyD88 pro-inflammatory pathway, modulation of the E-cadherin/β-catenin complex, and immune evasion through the Fap2–TIGIT axis, which suppresses NK and T-cell cytotoxic activity. These mechanisms help explain the ability of *F. nucleatum* to persist within tumor tissue and contribute to a pro-inflammatory, immunosuppressive microenvironment. For Pg, orthotopic and genetic models indicate activation of the NLRP3 inflammasome and recruitment of myeloid cells, with downstream amplification of TLR4/NF-κB signaling and release of TNF-α/IL-6/IL-8, favoring proliferation, angiogenesis, and immune evasion [22,49,50]. Notably, Fn–Pg co-occurrence may yield supra-additive inflammatory responses and transcriptional programs that converge on survival and anti-apoptotic pathways [23,51,52], outlining a plausible microbial consortium within the tumor niche. Our integrated view is further supported by tissue-level and host-level signals. On the tissue side, Fn enrichment in tumors versus adjacent mucosa and links with nodal metastasis provide biological anchoring between microbe and neoplastic tissue [17]. On the host side, prospective cohorts relating anti-periodontal IgG patterns to CRC risk [32] and case–control studies connecting oral microbiome profiles to CRC history [29] collectively suggest that long-term exposure to oral pathobionts may mirror or promote a procarcinogenic inflammatory state. Administrative datasets also point to associations between periodontitis and early colorectal neoplasia, albeit with variable precision [31]. Within the Fn subgroup (Table 2), tissue-based evidence is especially consistent for prognosis and molecular features. Higher tumoral Fn by qPCR associates with poorer CRC-specific survival and with MSI+/BRAF-mutated phenotypes [35,36], compatible with a more aggressive biology. Fecal Fn appears more promising for diagnosis/screening than for prognosis: population-based and multicenter studies show improved CRC discrimination using Fn (±Parvimonas micra, Peptostreptococcus) in stool-based panels [34,53]. It is also important to note that some primary studies reported partial or internally matched control groups, which may explain small inconsistencies in sample sizes across tables. These variations do not alter the direction of the results but reduce comparability between studies and should be considered when interpreting the pooled findings. However, the clinical application of *F. nucleatum* as a screening biomarker remains limited by the absence of standardized diagnostic thresholds and variability in sensitivity and specificity across studies. Some multicenter cohorts suggest that incorporating *F. nucleatum* into fecal immunochemical testing (FIT) panels may increase the detection accuracy for advanced neoplasia, but evidence remains preliminary and lacks harmonized cut-off values. Future translational studies should prioritize defining quantitative thresholds, validating sensitivity/specificity ranges, and evaluating the incremental predictive value of combining *F. nucleatum* with FIT in real-world screening programs. Anatomical distribution and microbial consortia also matter: several studies report higher Fn frequency in proximal colon and functional cooperation with other oral taxa in invasion/migration models [37,38]. For Pg, tissue and stool detection is less prevalent but potentially more phenotype-specific. In U-CAN/FECSU cohorts, Pg was associated with MSI tumors, stool–tissue concordance, and worse cancer-specific survival [21]. Case–control work found Pg in cases but not controls, frequently co-occurring with Fn/Prevotella [42]. Translationally, human and murine evidence suggests that Pg burden correlates with worse OS/RFS and that Pg-driven tumor promotion depends on hematopoietic NLRP3 signaling [22]. In vitro and MAM studies reinforce mechanisms of epithelial adhesion, MAPK/ERK activation, and immune evasion via CHI3L1-mediated iNKT inactivation [23,24,25,49]. Altogether, Pg may help define a microbe-influenced CRC subtype with diagnostic and prognostic implications [54].

### 4.1. Strengths and Limitations

A key strength of this study is the triangulation of epidemiological risk, cross-matrix microbiology (tumor and stool), and prognosis, analyzed with random-effects models, heterogeneity metrics, prediction intervals, and influence checks. Nonetheless, several limitations temper direct clinical translation: (i) between-study heterogeneity in periodontitis definitions, detection methods (qPCR/16S/IHC), specimen type, and confounder adjustment; (ii) the predominance of observational designs with potential residual confounding (e.g., smoking, antibiotics, colonoscopy screening); (iii) limited power for small-study bias tests in some subgroups; and (iv) variability in microbial quantification and cutoffs. Additionally, wide prediction intervals in some analyses reflect substantial between-study variability and highlight that, in some populations, the true effect may range from minimal to clinically meaningful. These broad intervals arise from differences in sample definitions, control selection, and reporting completeness in several included studies. Furthermore, differences in microbial detection protocols across studies—including variation in qPCR platforms, 16S rRNA sequencing depth, immunohistochemical thresholds, and analytical cutoffs—may affect cross-study comparability. Standardized detection methods will be essential in future investigations to improve methodological consistency and reproducibility. In addition, the number of available studies—particularly for the periodontitis–CRC association (*n* = 5)—was insufficient to support quantitative assessment of confounding or meaningful sensitivity analyses, limiting our ability to fully evaluate the stability of the results. Moreover, although we explored study-level characteristics and computed prediction intervals, the small number of available studies per comparison (k ≤ 5) did not permit a reliable meta-regression to further partition the sources of heterogeneity. Accordingly, effect sizes should be interpreted as signals of association, not proof of causality.

Although this systematic review and meta-analysis identified consistent associations between the presence of oral pathogens—particularly *Fusobacterium nucleatum* and *Porphyromonas gingivalis*—and colorectal cancer, these findings do not establish a causal relationship. The observed associations may reflect indirect mechanisms mediated by systemic inflammation, immune modulation, or shared risk factors rather than direct bacterial carcinogenesis. Therefore, causality cannot be inferred from the available observational and clinical data, and further mechanistic and longitudinal studies are warranted.

### 4.2. Clinical and Research Implications

Clinically, periodontal assessment and stool-based microbial markers (e.g., Fn ± co-pathogens) may serve as auxiliary tools to refine risk stratification and prioritize colonoscopy in selected subgroups, without replacing established screening pathways. In CRC patients, tumoral Fn/Pg burden could inform prognostic stratification and guide eligibility for trials targeting microbial–immune axes (e.g., FadA/gingipains, NLRP3/TLR4–NF-κB). Future research should prioritize well-powered prospective cohorts, standardized microbial assays and cutoffs, and mechanistic/therapeutic studies testing whether modifying oral inflammation or specific microbial pathways alters CRC outcomes.

## 5. Conclusions

Our findings clearly show that oral health and microbiome balance play a deeper role than traditionally recognized. Periodontitis and the presence of bacteria such as *Fusobacterium nucleatum* and *Porphyromonas gingivalis* not only affect oral tissues but are also associated with a higher risk of developing colorectal cancer and with poorer survival among those who have the disease. Trial sequential analysis confirmed that this relationship is consistent, robust, and statistically conclusive, reinforcing the concept that the oral–gut axis is a key biological pathway in colorectal carcinogenesis. These results remind us that maintaining good oral health is not merely an aesthetic or functional concern, but an essential component of cancer prevention. Understanding how oral bacteria influence inflammatory and tumor processes opens new opportunities for research, early diagnosis, and the design of more comprehensive, preventive therapeutic strategies. Nevertheless, these findings reflect associations rather than causality. The available evidence does not establish a direct bacterial etiology for colorectal cancer, and the observed links may instead represent indirect or context-dependent mechanisms. Future longitudinal and experimental studies are needed to elucidate the causal pathways underlying the oral–gut axis in colorectal carcinogenesis.

## Figures and Tables

**Table 1:** Characteristics and main findings of studies included in the meta-analysis on oral pathogens, periodontitis, and colorectal cancer risk.

Author (Year)	Country/Cohort	Study Design	N (Total)	CRC Cases	Controls	Exposure/Detection Method	Tumor Type/Sample	HR (95% CI)	Main Conclusion
Castellarin et al., 2012 [17]	Canada—BC Cancer Agency Tumor Repository	Case–control (tumor tissue vs. healthy tissue)	198	99	99	Detection of *Fusobacterium nucleatum* DNA by qPCR and RNA-seq	Colorectal tissue	3.58 [1.15–11.16]	*F. nucleatum* was ~415× more abundant in tumor tissue and was associated with lymph node metastasis.
Kato et al., 2016 [29]	USA—Detroit Metropolitan Area	Population-based case–control	190	68	122	16S rRNA sequencing of the oral microbiome (mouth rinse)	Self-reported CRC history	2.08 [1.08–4.00]	Higher abundance of *Lactobacillus* and *Rothia* in participants with a history of CRC; potential role of oral dysbiosis.
Lund Håheim et al., 2022 [30]	Norway—Oslo Cohort	Prospective cohort (17.5-year follow-up)	621	26	595	Serum IgG antibodies against *T. denticola, T. forsythia, P. gingivalis, A. actinomycetemcomitans* (ELISA)	Colon cancer (ICD-10: C18)	1.52 [1.06–2.19]	Low antibody titers to *T. denticola* were associated with higher risk of colon cancer.
Fu et al., 2022 [31]	Taiwan—NHIRD	Population-based retrospective cohort (15 years)	35,124	3865	31,259	Clinical diagnosis of periodontitis (ICD-9-CM)	Benign and malignant colorectal tumors	1.32 [0.74–2.36]	Periodontitis was associated with higher risk of benign colorectal tumors; positive, non-significant trend for malignant tumors.
Debertin et al., 2023 [32]	USA—CLUE I Cohort (Maryland)	Nested prospective cohort (matched case–control)	400	200	200	Serum IgG antibodies against oral pathogens (*A. actinomycetemcomitans, P. gingivalis, F. nucleatum,* etc.)	Colon cancer	2.17 [1.12–4.21]	High immune response to *A. actinomycetemcomitans* doubled the risk of colon cancer.

**Abbreviations:** CRC = colorectal cancer; HR = hazard ratio; 95% CI = 95% confidence interval; qPCR = quantitative polymerase chain reaction; RNA-seq = RNA sequencing; 16S rRNA = 16S ribosomal RNA; DNA = deoxyribonucleic acid; IgG = immunoglobulin G; ELISA = enzyme-linked immunosorbent assay; ICD-10 = International Classification of Diseases, 10th Revision; ICD-9-CM = International Classification of Diseases, 9th Revision, Clinical Modification; NHIRD = National Health Insurance Research Database (Taiwan); USA = United States of America; BC Cancer Agency = British Columbia Cancer Agency; CLUE I Cohort = “Give Us a Clue to Cancer and Heart Disease” (Maryland Cohort, USA); *F. nucleatum* = *Fusobacterium nucleatum*; *P. gingivalis* = *Porphyromonas gingivalis*; A. actinomycetemcomitans = *Aggregatibacter actinomycetemcomitans*.

**Table 2:** Characteristics of studies included in the systematic review and meta-analysis on *Fusobacterium nucleatum* in colorectal cancer.

Author (Year)	Country (Cohort)	Study Design	N (Total)	CRC Cases	Controls	Exposure Method	Tumor Type	CRC Diagnostic Method	Conclusion
Xie et al. (2022) [33]	China	Retrospective observational (tumor tissue vs. adjacent)	368	184 CRC	184 adjacent (internal)	Immunohistochemistry (Fn and MSI)	Colon and rectum	Histopathology	Fn positivity predicts worse survival.
Eisele et al. (2021) [34]	Germany, USA	Prospective cohort (ColoCare)	105	105 CRC	0 (no external controls)	qPCR in stool (Fn DNA)	Colon vs. rectum	Histopathology	High fecal Fn associated with rectal tumor, not survival.
de Carvalho et al. (2019) [35]	Brazil	Retrospective cohort (tumor tissue vs. adjacent)	209	152 CRC	57 adjacent (internal)	qPCR in tumor tissue	Colon and rectum	Histopathology	Fn associated with MSI+, *BRAF* mutation, and worse survival.
Mima et al. (2016) [36]	USA (NHS + HPFS)	Prospective cohort	1069	1069 CRC	0 (no external controls)	qPCR in tumor tissue	Colon and rectum	Centralized histopathology	High Fn load associated with CRC-specific mortality.
Rye et al. (2022) [37]	Norway (ColoCare)	Prospective cohort (tumor/adjacent pairs)	112	56 CRC	56 adjacent (internal)	qPCR in tumor and adjacent tissue	Colon and rectum	Histopathology	Fn more frequent in tumor; association with proximal location.
Lo et al. (2022) [38]	Taiwan	Retrospective + molecular cohort	203	116 CRC	87 (35 adenomas + 52 normal mucosa)	16S rRNA + qPCR (*Prevotella*, Fn)	Colon and rectum	Histopathology	*P. intermedia* and Fn promote invasion and metastasis.
Mima et al. (2020) [39]	Japan (Kumamoto Univ.)	Retrospective surgical cohort (operated CRC)	256	256 CRC	0 (no external controls)	qPCR in fresh tumor tissue	Colon and rectum	Histopathology	High *Bifidobacterium* levels ↑ risk of anastomotic leakage; Fn not significant.
Yu et al. (2017) [40]	China, Denmark, France, Austria	Multicenter case–control + validation (fecal)	Discovery: 128 (74 CRC, 54 ctrls); Validation: 156 (47 CRC, 109 ctrls)	121 CRC (disc. + val.)	163 external controls (54 + 109)	Metagenomic sequencing + qPCR (Fn, *Parvimonas*, *Peptostreptococcus*)	Colon and rectum	Colonoscopy + histopathology	Fecal Fn and *Parvimonas micra* are robust biomarkers for CRC.
Chen et al. (2022) [41]	China (Qilu Hospital, Shandong Univ.)	Retrospective cohort + meta-analysis	141	141 CRC (63 LN+, 16 M1)	0 (within-CRC comparison)	qPCR in tumor tissue (Fn DNA)	Colon and rectum	Histopathology + TNM	High intratumoral Fn predicts nodal and distant metastasis.

CRC = colorectal cancer; Fn = *Fusobacterium nucleatum*; MSI = microsatellite instability; qPCR = quantitative polymerase chain reaction; 16S rRNA = 16S ribosomal RNA; DNA = deoxyribonucleic acid; IHC = immunohistochemistry; NHS = Nurses’ Health Study; HPFS = Health Professionals Follow-up Study; ColoCare = ColoCare cohort; BRAF = BRAF proto-oncogene; TNM = Tumor–Node–Metastasis staging; LN+ = lymph node–positive; M1 = distant metastasis present; USA = United States of America; Univ. = University; ctrls = controls; disc. = discovery; val. = validation; vs. = versus; ↑ = increased.

**Table 3:** Characteristics of studies assessing the association between *Porphyromonas gingivalis* and colorectal cancer.

Author (Year)	Country/Cohort	Study Design	N (Total)	CRC Cases	Controls	Exposure/Detection Method for *P. gingivalis*	Tumor Type/Model	CRC Diagnostic Method	Main Conclusion
Kerdreux et al., 2023 [21]	Sweden—U-CAN and FECSU cohorts	Cross-sectional (CRC, dysplasia, and controls)	563	285 (CRC: 247 U-CAN + 38 FECSU)	150 (89 U-CAN + 61 FECSU)	qPCR in stool and tumor tissue for *P. gingivalis*	Colorectal adenocarcinoma (MSI/MSS; mucinous/non-mucinous)	Clinical and histopathological within institutional cohorts	*P. gingivalis* detected in 2.6–5.3% of cases; associated with MSI tumors and lower cancer-specific survival (*p* = 0.04).
El-Sokkary, 2022 [42]	Egypt—Mansoura Oncology Hospital	Case–control (stool PCR)	34	27	7	Conventional PCR and qPCR for 11 bacterial species, incl. *P. gingivalis*	Clinically diagnosed CRC	Hospital clinical diagnosis + histopathological confirmation	*P. gingivalis* detected only in cases (0% in controls); co-occurs with *Fusobacterium* and *Prevotella*. Potential fecal biomarker.
Wang et al., 2021 [22]	China—Sun Yat-sen University Hospital	Translational: case–control (CRC vs. adenoma vs. healthy) + prognostic cohort (two CRC cohorts)	>400 humans (392 in CRC cohorts + case–control group)	392	NR (adenoma + healthy)	qPCR and immunohistochemistry in tissue and stool; oral infection in murine models	Human CRC; orthotopic models and Apc^Min/+^	Histology + TNM staging	*P. gingivalis* enriched in CRC vs. adenoma/healthy; high tumor burden → worse OS/RFS (HR ≈ 2.3–2.6). Promotes CRC via hematopoietic NLRP3 inflammasome.

CRC = colorectal cancer; qPCR = quantitative polymerase chain reaction; PCR = polymerase chain reaction; IHC = immunohistochemistry; MSI/MSS = microsatellite instability/stability; TNM = Tumor–Node–Metastasis staging; OS = overall survival; RFS = recurrence-free survival; HR = hazard ratio; NR = not reported; vs. = versus; incl. = including; *P. gingivalis* = *Porphyromonas gingivalis*; Apc^Min/+^ = APC “multiple intestinal neoplasia” mouse model; U-CAN = Swedish U-CAN cohort (proper name); FECSU = Swedish FECSU cohort (proper name).

**Table 4:** (CRC risk from periodontitis/oral exposure)—ROBINS-I (summary).

Study	Confounding	Exposure Classification	Outcome Measurement	Overall Risk
Castellarin 2012 [17]	Some concerns	Low	Low	Some concerns
Kato 2016 [29]	Some concerns	Some concerns	**High** (self-reported CRC)	**Some/High**
Lund Håheim 2022 [30]	Some concerns (serology as proxy)	Some concerns	Low	Some concerns
Fu 2022 [31]	**High** (limited adjustment)	Some concerns (ICD)	Some concerns	**High**
Debertin 2023 [32]	Some concerns	Some concerns (IgG panel)	Low	Some concerns

**Table 5:** (*Fusobacterium nucleatum* in CRC)—QUADAS-2/QUIPS (summary).

Study	Selection	Index Test (qPCR/16S/IHC)	Confounders/Analysis (If Prognostic)	Overall Risk
Xie 2022 [33]	Some concerns	Some concerns (IHC variability)	Some concerns	Some concerns
Eisele 2021 [34]	Some concerns (cases only)	Some concerns (fecal thresholds)	**Low**	Some concerns
de Carvalho 2019 [35]	Some concerns	**Low** (tissue qPCR)	Some concerns	Some concerns
Mima 2016 [36]	**Low**	**Low** (tissue qPCR)	**Low** (adjusted Cox)	**Low**
Rye 2022 [37]	**Low** (tumor/adjacent pairs)	**Low** (qPCR)	**Low**	**Low**

**Table 6:** (*Porphyromonas gingivalis* in CRC)—QUADAS-2/QUIPS/JBI (summary).

Study	Selection	Pg Detection (qPCR/PCR/IHC)	Confounders/Analysis (If Prognostic)	Overall Risk
Kerdreux 2023 [21]	**Low**	Some concerns (low prevalence, LOD)	Some concerns (CSS, MSI)	Some concerns
El-Sokkary 2022 [42]	**High** (small sample, hospital controls)	Some/High	**Low**	**High**
Wang 2021 [22]	Some concerns	**Low** (concordant qPCR/IHC)	**Low** (Cox adjusted for TNM)	**Low**

## Data Availability

Dataset available on request from the authors.

## Supplementary Materials

The following supporting information can be downloaded at: https://www.mdpi.com/article/10.3390/dj13120595/s1, Table S1: PRISMA_2020_Checklist.
